# Metabolite Profiles of Male and Female Humboldt Penguins

**DOI:** 10.3390/vetsci2040349

**Published:** 2015-10-14

**Authors:** Jeffrey M. Levengood, David J. Schaeffer, Alexander V. Ulanov

**Affiliations:** 1 Department of Comparative Biosciences, College of Veterinary Medicine, University of Illinois at Urbana-Champaign, Urbana, IL 61802, USA; E-Mail: ecohealth@aol.com; 2 Metabolomics Center, Roy J. Carver Biotechnology Center, University of Illinois at Urbana-Champaign, Urbana, IL 61801, USA; E-Mail: ulanov@illinois.edu

**Keywords:** Humboldt Penguin, *Spheniscus humboldti*, metabolome, gender, 2-oxoglutarate, glycerol 3-phosphate

## Abstract

We examined 185 metabolites in 30 adult Humboldt Penguins (*Spheniscus humboldti*) nesting at the Punta San Juan Marine Protected Area, Peru, in order to examine gender differences in metabolome profiles, particularly those involved in metabolism and energetics. The majority of the compounds identified were fatty (26% of total identified compounds), organic (19%), and amino (16%) acids. We were able to differentiate male and female penguins with 96.6% accuracy on the basis of 12 metabolites, most of which are involved in lipid and carbohydrate metabolism. These included 2-oxoglutarate, erythronic acid, GABA, mannitol, sedoheptulose 7-phosphate, and serine and six metabolites present in higher concentrations in females compared to males (2-aminoadipic acid, *O*-phosphorylethanolamine, glycerol 2-phosphate, glycerol 3-phosphate, pantothenic acid, and creatinine). Of these, 2-oxoglutarate and glycerol 3-phosphate were key metabolites distinguishing gender. Our results indicated that male and female Humboldt Penguins were characterized by differing metabolic states. Such differences could be important to individual and brood survival in times of environmental stress.

## 1. Introduction

The Humboldt Penguin (*Spheniscus humboldti*) is endangered throughout its range (southern Peru to northern Chile) due to a variety of external population pressures [1]. Anthropogenic impacts have led to a smaller population that is less robust to the impact of El Niño Southern Oscillation (ENSO) events. The Punta San Juan Marine Protected Area (PSJMPA) contains the largest nesting colony of Humboldt Penguins in Peru. Humboldt Penguins are a burrow-nesting, relatively shallow-diving species that have a prolonged breeding season (March-December) in Peru, and can have two clutches during that time [2]. Male Humboldt Penguins are slightly larger, on average, than females [3], and although one study reported that the foraging behaviors of the two sexes were similar in relation to foraging time and effort [4], a more recent study [5] found that the total distance traveled by males was greater than for females; total time for foraging trips did not differ between sexes but males dived deeper and maximum diving efficiency was greater in females. Humboldt Penguins cannot forage during molt, which starts earlier and takes longer in males than in females [6].

*Metabolomics* is an area of systems biology in which changes in the metabolome, or profile of metabolites of low-molecular weight, is examined in relation to some stimuli or perturbation. No systems-level studies of wild birds been reported. Some studies have examined smaller numbers of metabolites, which have revealed that some metabolites are reflective of energetic changes (*i.e.*, as revealed in mass loss) over the short-term (days), (e.g., for Western Sandpipers (*Calidris mauri*, [7]; Lesser Scaup (*Aythya affinis*) [8], and even longer-term (months, e.g., King Penguins [9].

Across classes, males and females have different mechanisms supplying biosynthesis pathways and generation of cellular energy due to differences in biosynthesis needs. We hypothesized that published differences in the energy balances of male and female animals [9,10,11,12,13,14,15,16,17] would be associated with uncharacterized dimorphic differences in metabolite profiles encompassing a broad range of metabolome classes. It is reasonable to expect subtle metabolic differences between genders arising from overt differences in foraging behavior and energetic parental investment in this species. Herein we describe the untargeted environmental metabolomics (*sensu* Bundy *et al.* [18]) of previously molecularly-sexed male and female Humboldt Penguins during a portion of the breeding season.

## 2. Experimental Section

### 2.1. Sample Collection and Processing

Fieldwork was conducted within the Punta San Juan Marine Protected Area, Ica, Peru (15°22ʹ S, 75°12ʹ W) during 2009. Samples were collected in May, which typically coincides with one of two annual peaks in egg-laying in this population. Blood was collected in conjunction with ongoing health assessments of this population by veterinarians (Michael Adkesson, Jennifer Langan) with the Chicago Zoological Society’s Brookfield Zoo. Methods were approved by the Saint Louis Zoo Institutional Animal Care and Use Committee, Protocol Number 09–02 and the Peruvian Ministry of Agriculture. Adult penguins were manually restrained and removed from the nest. Time from approach to capture and blood collection was similar in all penguins. Capture, restraint, and sample collection was performed quickly and with as little stress as possible. Penguins were examined by a veterinarian and deemed to be in normal health based on physical examination. A 20 ga needle was used to collect up to 24 cc of blood from the jugular vein of penguins. Gender was determined by molecular methods following Wallace *et al.* [19].

Samples were maintained on icepacks until processing and serum was separated within 4–6 h following collection. Samples were placed into cryovials (NUNC, Thermo Fisher Scientific, Rochester, New York, NY, USA) and frozen to −20 °C. Within 1–5 days, samples were placed in liquid nitrogen and for transport and maintained at −80 °C until analysis.

### 2.2. Metabolite Profiling

There are many published metabolomic studies of human blood plasma or serum fractions, e.g., [20,21,22]. However, with the exception of the reagents used for derivatization, no standardized, universally-accepted method currently exists. Our analytical methods closely followed Jiye *et al.* [22] and are detailed below.

Methanol and water were HPLC grade (EMD chemicals Inc., Temecula, CA, USA). Pyridine, MSTFA, chloroform, diazomethane, methoxyamine hydrochloride, and all standards were Sigma-Aldrich (Saint Louis, MO, USA). For volatile compounds analysis, 100 μL of blood was mixed with 25 μL of methanol for protein precipitation, centrifuged for 5 min at 11,000 g (Beckman Microfuge 18, Beckman Coulter Inc., Brea, CA, USA) and 20 μL of the internal standard methyl phthalate (0.5 mg/mL) were applied to the collected supernatants. A 5 μL aliquot was injected into a GC/MS system (Agilent Inc., Palo Alto, CA, USA) consisting of a Agilent 7890 gas chromatograph, an Agilent 5975 mass selective detector, and a HP 7683B in splitless mode (0.5 min). Metabolite separation was performed on a ZB-WAX capillary column (30 m × 0.32 mm I.D. and 0.25 µm film thickness) (Phenomenex, Torrance, CA, USA). The helium carrier gas was kept at a constant flow rate of 2.7 mL·min^−1^. The inlet and MS interface temperatures were 250 °C, and the ion source temperature was adjusted to 230 °C. The temperature program was initial 5-min isothermal heating at 50 °C, followed by an oven temperature increase of 5 °C·min^−1^ to 265 °C and a final 5 min at 265 °C. The mass spectrometer was operated in positive electron impact mode (EI) at 69.9 eV ionization energy in *m*/*z* 30–800 scan range.

For metabolite profiling and free fatty acid analyses, blood samples were extracted as follow: Each sample (400 μL blood) was threefold extracted (1 min vortex) with 1.5 mL of methanol:water:chloroform (2:1:1) and centrifuged at 11,000 g for 10 min. Polar and non-polar phases were collected separately and dried under a N_2_ stream and subjected to metabolite profiling and free fatty acid lysis correspondingly.

For metabolic profiling, dried polar extracts were derivatized with 80 μL methoxyamine hydrochloride (20 mg·mL^−1^) for 60 min at 50 °C and then with 80 μL MSTFA at 70 °C for 120 min with a following 2-h incubation at room temperature. Ten microliters (10 μL) of the internal standard (hentriacontanoic acid, 10 mg/mL) was added to each sample prior to derivatization. Samples were analyzed on a GC/MS system (Agilent Inc., Palo Alto, CA, USA) consisting of an Agilent 7890 gas chromatograph, an Agilent 5975 mass selective detector, and a HP 7683B autosampler. Gas chromatography was performed on a HP-5MS (60 m × 0.25 mm I.D. and 0.25 µm film thickness) capillary column (Agilent Inc., Palo Alto, CA, USA). The inlet and MS interface temperatures were 250 °C, and the ion source temperature was adjusted to 230 °C. An aliquot of 1 µL was injected with the split ratio of 10:1. The helium carrier gas was kept at a constant flow rate of 1.5 mL·min^−1^. The temperature program was: 5-min isothermal heating at 70 °C, followed by an oven temperature increase of 5 °C·min^−1^ to 310 °C and a final 10 min at 310 °C. The mass spectrometer was operated in positive electron impact mode (EI) at 69.9 eV ionization energy in the *m*/*z* 30–800 scan range.

Free fatty acid analysis from non-polar extracts were converted to their methyl esters and analyzed with the same instrument on a ZB-WAX capillary column (30 m × 0.32 mm I.D. and 0.25 µm film thickness) (Phenomenex, Torrance, CA, USA) with an injection port temperature of 260 °C, the interface set to 280 °C, and the ion source adjusted to 230 °C. The helium carrier gas was set at a constant flow rate of 3 mL·min^−1^. The temperature program was 5-min isothermal heating at 140 °C, followed by an oven temperature increase of 10 °C·min^−1^ to 265 °C for a final 25 min. The mass spectrometer was operated in positive electron impact mode (EI) at 69.9 eV ionization energy in the *m*/*z* 30–800 scan range.

The spectra of all chromatogram peaks were compared with electron impact mass spectrum libraries NIST08 (NIST, Gaithersburg, MD, USA), W8N08 (John Wiley and Sons, Inc, Hoboken, NJ, USA), and a custom-built library. To allow comparison between samples, all data were normalized to the internal standards in each chromatogram and the bird’s weight. The spectra of all chromatogram peaks were evaluated using the HP Chemstation (Agilent, Palo Alto, CA, USA) and AMDIS (NIST, Gaithersburg, MD, USA) programs. Validation of extraction, derivatization, and GC-MS analysis was performed for selected metabolites of different classes: sugars (mono- and disaccharides, organic acids, amino acids, alcohols, fatty acids) and “recovery” percentage was calculated [23]. Metabolite concentrations were calculated as (analyte concentration relative to hentriacontanoic acid) per 1.0 mL blood (relative concentration), *i.e.*, as target compound peak area divided by the internal standard (IS) peak area (IS concentration is the same in all samples). Hentriacontanoic acid (C_31_H_62_O_2_) is a fatty acid that is usually absent. It is not possible to build calibration curves for all identified metabolites especially as some of them are not commercially available as pure standards. Relative concentration (RC) is commonly used to compare individual and suites of metabolites between samples [24,25,26].

### 2.3. Quantification

Peak areas, X, are normalized to the fresh weight of the sample and response of the internal standard, hentriacontanoic acid (C_31_H_62_O_2_), a fatty acid that is usually absent in any real sample. The normalized concentration is N_i_ = X_i_ × X^−1^_hentriacontanoic acid_ × fresh·weight^−1^. Then, the relative response of a fragment, R_i_, is divided by the average normalized responses of all samples in one experiment or of all non-treated control samples of an experiment: R_i_ × N_i_ × *avg*N^−1^. The complete set of metabolite response ratios of all samples is combined into a single matrix that describes an experiment or group of experiments [27,28].

The instrument variability was 5% that is within the standard acceptance limit. Chemometric models were obtained using log-transformed and autoscaled data. Multiple comparisons were accounted for with the False Discovery Rate (FDR) method [29]. Obtained metabolite names were converted into KEGG IDs and mapped using the *Gallus gallus* (chicken) database with Metaboanalyst 2.0 (http://www.metaboanalyst.ca). 

To examine whether gender affects the blood metabolome we analyzed blood samples from 12 male and 18 male penguins. Metabolite levels were calculated by comparison of the ion features in the experimental samples to a reference database of chemical standard entries with following normalization to the internal standard. Log-transformation was applied to correct for heteroscedasticity, to convert multiplicative relations into additive relations, and to make skewed distributions (more) symmetric; mean-centering or autoscaling were applied in order to remove the overall offset [30,31]. Moreover, autoscaling avoids the possibility that a few high-intensity variables dominate the final solution. Metabolites with 30% and more of missing data were removed from analysis; the rest of missing values for particular metabolite were imputed with the observed minimum detection value, assuming their level was below the instrument detection limit.

Twenty two metabolites with large analytical variations were omitted from the analysis to avoid the correlation interference in further statistical analysis. All spurious metabolites derived from column bleed, reagent artifacts and xenobiotics were also removed from the data sets. One sample (male) has been omitted from the analysis as an outlier. We compared mean relative concentrations using one-way ANOVA with Bonferroni *post hoc* testing. A probability level of *p* ≤ 0.05 was considered statistically significant.

## 3. Results

### 3.1. GC/MS Identification of Metabolites

GC/MS analysis of both polar and non-polar metabolites detected a total of ~1300 EI mass spectral features in the samples from 30 penguins. Of these, 185 compounds were positively identified with the majority being fatty acids (26% of total identified compounds). Other metabolites included organic acids (19%), amino acids, and nucleotides (16% and 6% correspondingly), carbohydrates (11%), alcohols (10%) and other metabolite classes (10%).

### 3.2. Gender Discrimination of Metabolite Concentrations

To analyze the overall levels of identified metabolites within and between subjects, a Box-plot analysis of metabolite levels was performed. All samples showed an acceptable range of metabolite levels and degree of variation within each cohort. We identified total of 12 metabolites distinguishing males and females across both cohorts (*p* ≤ 0.05): 6 compounds with higher concentrations in males compared to females (2-oxoglutarate, erythronic acid, GABA, mannitol, sedoheptulose 7-phosphate, and serine) and 6 metabolites that were higher in females compared to males (2-aminoadipic acid, *O*-phosphorylethanolamine, glycerol 2-phosphate, glycerol 3-phosphate, pantothenic acid, and creatinine). Lacking published data for these compounds in penguins (other than creatinine), Table 1 gives metabolic roles for each compound in vertebrates.

**Figure 1:**
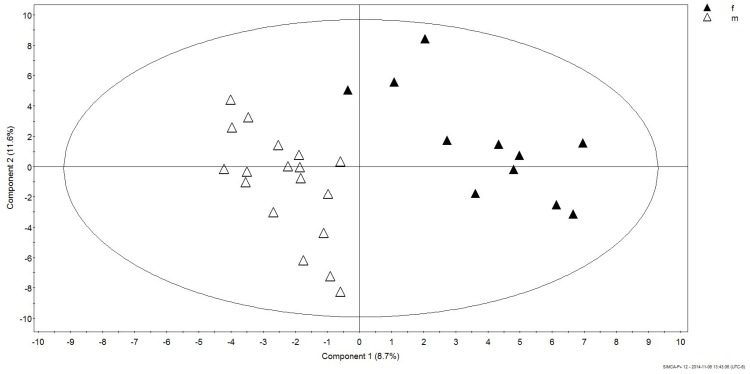
PLS-DA score plot obtained for male and female Humboldt Penguins.

**Figure 2:**
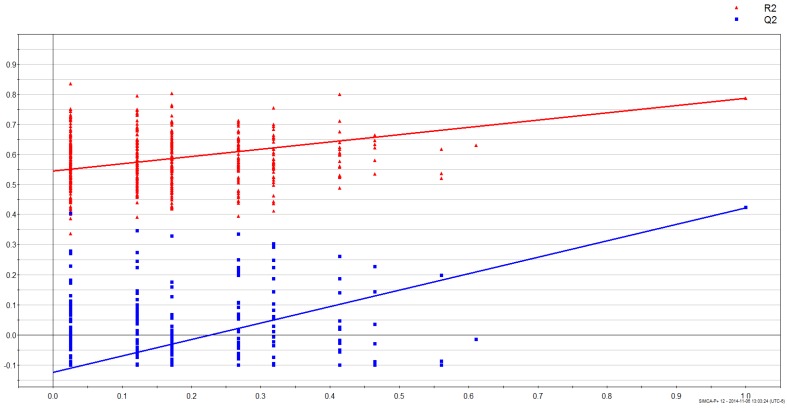
Results from response permutation test of gender (male *vs.* female) samples. The vertical axis gives the R2 (red) and Q2 (blue)-values of the original model (far to the right) and the Y-permuted models further to the left. The horizontal axis shows the correlation between the permuted y-vectors and the original y-vector for the selected y.

Maximum separation of data according to gender was achieved in a PLS-DA score plot (PC1 *versus* PC2) with UV-scaling (Figure 1). The validity of the obtained PLS-DA models were further evaluated using an analysis of variance of sevenfold Cross-Validation predictive residual (CV-ANOVA) and response permutation with 500 random reclassifications (random assignment of class labels to male and female in order to test whether differences found between groups are significant). Cross-Validation (CV) was used to determine the sufficient number of Principal Components (PCs) represented by the total amount of explained X variance (R2X), Y-variance (R2Y) and cross-validated predictive ability (Q2Y). In our case the PLS-DA model for gender had R2Y = 88.6%, Q2 = 31.6% and the variables explained 20.6% (R2X) of total variation. A permutation test demonstrates that the goodness of fit and predictive ability (R^2^/Q^2^) of the original models discriminating male from female was higher than those of the permuted models (Figure 2).

**Table 1:** Penguin metabolites with statistically significant gender dimorphism and example metabolic roles in vertebrates.

Penguin Metabolite	CASRN/HMDB ^1^	Example KEGG ^2^ (Map) and SMPDB ^3^ Pathways
2-aminoadipic acid	542-32-5 HMDB00510	Lysine degradation (SMP00037)
2-oxoglutarate	328-50-7 HMDB00208	2-oxocarboxylic acid metabolism (map01210), citrate (TCA) cycle (map00020), Glyoxylate and dicarboxylate metabolism (map00630)
creatinine	60-27-5 HMDB00562	Arginine and proline metabolism (map00330), numerous metabolic pathways (map01100)
erythronic acid	13752-84-6 HMDB00613	a sugar component of aqueous humour (eye) (HMDB00613)
GABA (gamma-aminobutyric acid)	56-12-2 HMDB00112	Alanine, aspartate and glutamate metabolism (map00250), cAMP signaling pathway (map04024)
Glycerol 2-phosphate	17181-54-3 HMDB02520	glycerolipid metabolism (HMDB02520)
Glycerol 3-phosphate	57-03-4 HMDB00126	glycolysis metabolic pathway (HMDB00126), glycerol phosphate shuttle (SMP00124), mitochondrial electron transport chain (SMP00355), glycerolopid metabolism (SMP00039), phospholipid biosynthesis (SMP00025)
mannitol	69-65-8 HMDB00765	Fructose and mannose metabolism (map00051), Phosphotransferase system (PTS) (map02060)
*O*-phosphorylethanolamine	1071-23-4 HMDB00224	Arginine and Proline Metabolism (SMP00020), Lactose Synthesis (SMP00444), Tryptophan metabolism (SMP00063), Cysteine metabolism (SMP00013), Mitochondrial Beta-Oxidation of Long Chain Saturated Fatty Acids (SMP00482), Mitochondrial Beta-Oxidation of Short Chain Saturated Fatty Acids (SMP00480)
pantothenic acid	79-83-4 HMDB00210	Antidyslipidemic agents (map07052), Vitamin digestion and absorption (map04977)
sedoheptulose 7-phosphate	2646-35-7 HMDB01068	Lipopolysaccharide biosynthesis (map00540), Carbon metabolism (map01200), Biosynthesis of amino acids (map01230)
succinate	110-15-6 HMDB00254 (succinic acid)	2-Oxocarboxylic acid metabolism (map01210), Biosynthesis of secondary metabolites (map01110), Alanine, aspartate and glutamate metabolism (map00250), Glyoxylate and dicarboxylate metabolism (map00630), citrate (TCA) cycle (map00020)

**^1^** The Human Metabolome Database; **^2^** Kyoto Encyclopedia of Genes and Genomes; **^3^** The Small Molecule Pathway Database.

### 3.3. Gender Differences in Metabolites

Following common statistical procedures to verify discriminant analysis results, we verified that the 12 metabolites correctly classified samples as male or female using a Random Forest (resampling) analysis. The 12 metabolites had a 96.6% predictive accuracy. All eighteen male samples and 10 of 11 female samples were correctly classified to gender. Thus, in accord with our hypothesis, metabolites associated with mechanisms supplying biosynthesis pathways and generation of cellular energy due to differences in biosynthesis needs, are differentially expressed between males and females. These metabolites are potential biomarkers for monitoring and quantifying gender-associated responses to stress in Humboldt Penguins.

**Figure 3:**
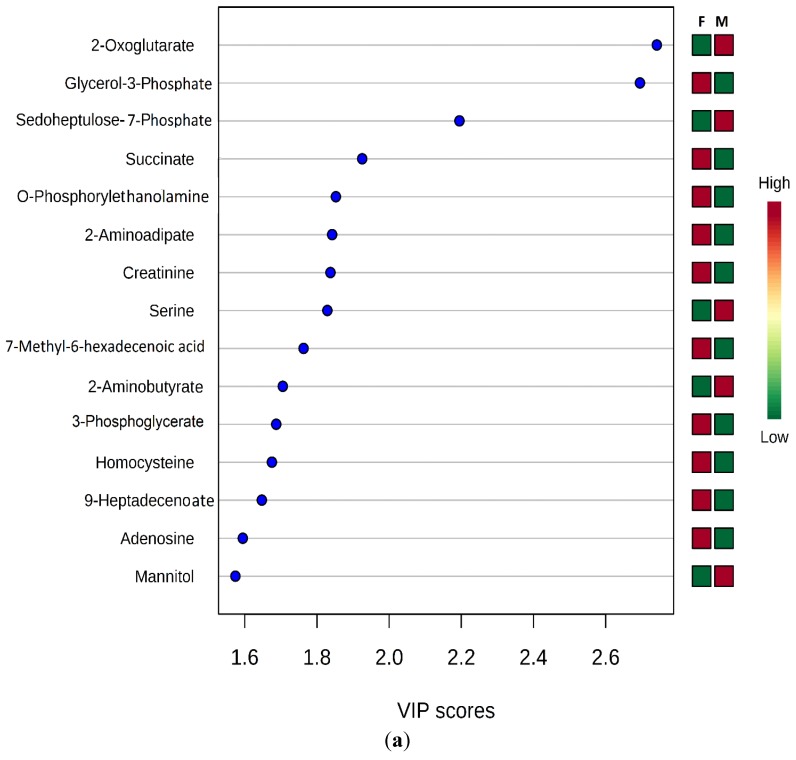

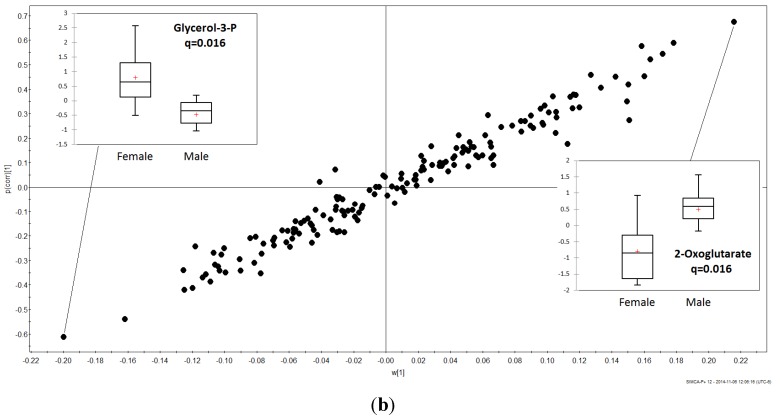
PLS-DA VIP plot (**a**); and OPLS-DA s-Plot (**b**) for male and female Humboldt Penguins.

Determination of the discriminating metabolites toward the clustering in PLS-DA models was further analyzed using regression coefficient plot with 95% jackknifed confident intervals, where metabolites with Variable Importance for Projection (VIP) values exceeding 1.0 were selected as metabolite cut off [32] and S-plot of the PLS-DA model (R2X = 28.0, R2Y = 94.7%, Q2 = 48.7%) (Figure 3a).

The statistical significance of metabolites with VIP value greater than one was verified by performing unpaired *t* test and Welch’s *t* test with following false discovery rate (FDR) control to address the problem of multiple comparisons and q-values were calculated for all identified metabolites. Among these, 2-oxoglutarate (2Ox) and glycerol 3-phosphate (G3P) had the lowest FDR values (*q* = 0.016). Males had greater mean 2Ox levels, whereas females had, on average, greater G3P levels (Figure 3b). The mean G3P concentration was higher in female than male Humboldt Penguins whereas the mean 2Ox concentration was higher in males than females (Figure 3b).

## 4. Discussion

Across classes, males and females have different mechanisms supplying biosynthesis pathways and generation of cellular energy due to differences in biosynthesis needs. Gender differences in metabolism and energy homeostasis appear to allow human females to oxidize fat and conserve protein during (energy deficit) or (increased energy expenditure) [10,11,12]. Gender differences in energy balance are evident during caloric restriction (CR), exercise, and starvation [13]. During starvation, women have more rapid increase in FFA and ketones than men [14], and during prolonged physical activity women mainly use fat whereas carbohydrates and amino acids are of primary import in men [15]. Colom *et al.* [16] reported that, following prolonged caloric restriction, muscle of female rats had greater mitochondrial activity and capacity than did males, and surmised that this gave females greater ability to respond to energetically demanding periods. And, female mice were more metabolically active in the presence of metabolite substrates than were males [17].

Gender differences in metabolites reflective of energetics have been documented in some avian species. Migrating male wagtails (*Motacilla* sp.) had greater plasma triglyceride, and lower β-hydroxybutyrate, concentrations than did females [33]. Arizmendi-Mejia *et al.* [34] noted that in Cory’s Shearwaters (*Calonectris borealis*) fat deposition and protein catabolism began after migration but before egg-laying, with females beginning these processes sooner than males. In Gull-billed Terns (*Gelochelidon nilotica*), a species with only slight sexual dimorphism, females chicks had lower triglyceride levels than males in a colony where growth rates and fledging success were lower [35]. Pre-breeding female macaroni penguins (*Eudyptes chrysolophus*) after foraging had higher concentrations of some metals, lower concentrations of total cholesterol, and similar concentrations of creatinine than the pre-breeding males. Creatinine concentrations were lower in pre-breeding than in pre-moult females, but were higher in pre-moult males than in pre-breeding males. The authors suggested that differences in plasma metabolite concentrations between pre-breeding males and females reflected mobilization for egg formation [36]. In a subsequent study this group reported that the mean concentrations. of creatinine were lower in the late moult groups in both macaroni (*Eudyptes chrysolophus*) and gentoo (*Pygoscelis papua*) penguins [37]. Metabolites thus are useful in field studies of avian energetics and physiological condition. However, to our knowledge no studies have compared a comprehensive suite of metabolites to determine if male and female birds, penguins in particular, have differences in metabolite profiles associated with energy requirements.

Of the 12 metabolites differing between male from female Humboldt Penguins, 2-oxoglutarate and glycerol 3-phosphate (G3P) were key metabolites distinguishing gender. G3P is the product of the conversion of dihydroxyacetone phosphate (DHAP) by oxidation of 1 molecule of NADH to 1 molecule of NAD^+^ in the glycerol phosphate shuttle. G3P is converted back to DHAP in the mitochondria, thereby making NADH synthesized in the cytosol by glycolysis available to the oxidative phosphorylation pathway in the mitochondria to generate ATP. Ji *et al.* [38] noted that G3P was lower in fasted and insulin-deprived than in *ad libitum* fed chickens. Given that “the G3P shuttle constitutes a crossroad between lipid and carbohydrate metabolism as well as the main entry of glycerol into gluconeogenesis” [39], the potential importance of differential levels of G3P between male and female penguins cannot be underestimated and deserves further investigation.

2-Oxoglutarate is an important intermediate in the CAC that is produced by the hydrolysis of glutamine then deamination of glutamate, thereby generating a NADH equivalent of 2.5 ATP. This metabolite is also important in the malate-aspartate shuttle, whereby oxaloacetate is reduced by NADH in the intermembrane space, and is one of the most important nitrogen transporters in metabolic pathways. Thus, differences we observed may signify important metabolic variation between male and female Humboldt Penguins, which may reflect subtle differences in foraging or brood-rearing behavior.

Sexual dimorphism in birds has been reported for plasma or serum chemistry of seabirds including penguins [9,40,41,42]. Gender differences in the metabolism of penguins have correlates in the sexual dimorphism of their foraging and energy balance strategies, which are more apparent in sub-Antarctic/Antarctic breeding penguin species. Plasma metabolite concentrations in Macaroni Penguins arriving on land for breeding differed between sexes, with higher lipids and lower total protein lower in females [37]. During the guard period, female Adélie Penguins (*Pygoscelis adeliae*) made longer foraging trips, ranged further more frequently and consumed larger quantities of krill than males [43]. The metabolic expenditure for the foraging necessary to accumulate the depot fat and lean tissue lost while fasting during the reproductive period was greater in in male Adélie Penguins, with females expending more energy during the post-hatch period but using less energy during courtship and incubation [44]. In Emperor Penguins, *Aptenodytes forsteri*, differing patterns of plasma free fatty acids and β-hydroxybutyrate between sexes reflected the differing roles and physiological needs of the genders in the reproductive process [45].

In contrast to sub-/Antarctic penguins, the only significant foraging difference between sexes in Humboldt Penguins (*Spheniscus humboldti*) during chick rearing was a greater dive depth for males [4]. Rey *et al.* [5] compared the foraging behavior of Humboldt and Magellanic (*S. magellanicus*) penguins breeding in sympatry during the chick-rearing period. The average duration of foraging trips did not differ significantly between species or between sexes of each species. For both species, males travelled farther and made significantly longer dives than females, whereas females made more direct trips (less sinuous) than males. Although studies have shown that male and female Humboldt Penguins have similar foraging behaviors during the breeding season and share in parental duties [4,5] our results indicate that their energetics differ in subtle ways that could be critical in times of stress.

## 5. Conclusions

We were able to distinguish male and female Humboldt Penguins during the breeding season on the basis of differences in their serum metabolomes. The majority of metabolites important in differentiating gender in these Humboldt Penguins are primarily involved in lipid and carbohydrate metabolism, indicating subtle energetic gender differences that could be important in times of environmental stress, when reduced food availability reduces survival and productivity [46]. Gender-specific survival of chicks may occur even in monomorphic species (e.g., Gull-billed Terns [35]; Common Terns (*Sterna hirundo*) [47]) and could enhance the competitive ability and differential survival of one or the other gender, depending on conditions (e.g., timing of event relative to growth stage), during times of dramatic food shortage (as in ENSO events).
